# Supplementary material for: Full Sails against Cancer
Source: Int J Environ Res Public Health. 2022 Dec 10;19(24):16609. doi: 10.3390/ijerph192416609 (PMC9778763; doi:10.3390/ijerph192416609)

FONDAZIONE
TENDER TO
NAVE ITALIA
ONLUS

The travel route can change every year depending on the sea conditions and the needs of the groups participating in the initiative. The fun and recreational activities offered are complemented by maritime and laboratory activities that the participants carry out on board with the help of the crew and carers.

MANEUVERING THE SAILS

Maneuvering the ropes together with the sailors, the group opens the sails under the orders of the captain and the boatswain. Collaborating and having team spirit are the basis of this activity.

MANEUVERING AT THE HELM

Another of the seafaring activities carried out on board. Supported by a professional sailor, the kids take control of the ship by steering it.

KNOTS WORKSHOP

In this preparatory workshop for learning how to construct the framework of knots with the line, the group learns the usefulness of the main nautical knots and how to make them.

ASCENT TO THE SHORE: The ascent ashore involves climbing the foremast up to the first crow's nest. It is one of the most probing activities for the participant: to climb to the top as a method to get to know each other better.

Summer 2022 departure ports:

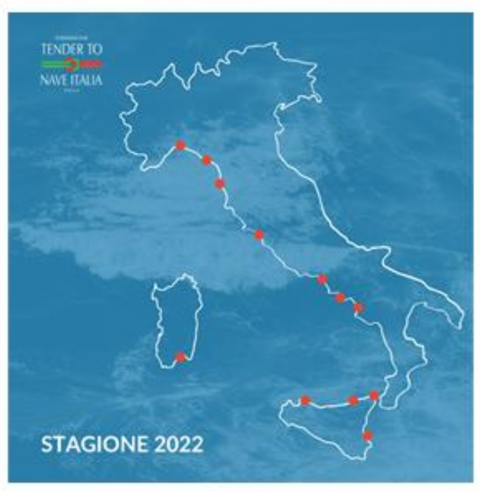

Supplement: Supplementary file 1 [file ijerph-19-16609-s001.zip › ijerph-1895588-supplementary.pdf]
